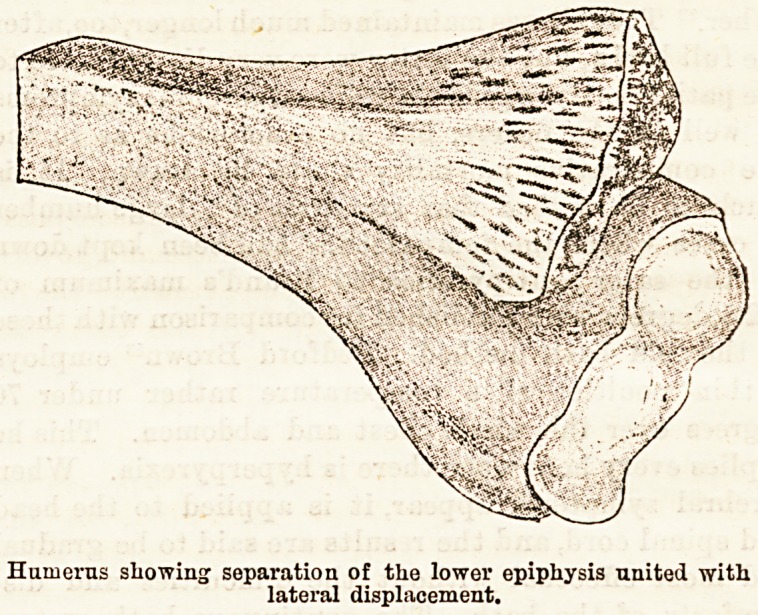# Progress in Surgery

**Published:** 1895-06-29

**Authors:** 


### Progress in Surgery.

### FRACTURES AND DISLOCATIONS.

**Massage and Mobilisation.**—The Lucas-Championnière treatment of fractures of the long bones by mobilisation comprises, says Brun,[1] procedures, which differ somewhat from each other: (1) The massage and gymnastics are begun immediately after the fracture, and are kept up continuously until recovery is complete from a functional point of view; (2) an unremovable apparatus is applied and allowed to remain for a few days before the massage and gymnastics are begun; (3) mobilisation and immobilisation of the fracture are resorted to in alternation (mixed treatment). Mobilisation is contra-indicated in cases in which considerable deformity with exaggerated mobility of the fractured ends exists, as well as in cases of oblique fracture with phlyctænæ, phlebitis, &c. The different modes of procedure in each particular fracture are fully detailed and figured in Lucas-Championnière's ([2]) recent volume, "Traitment des Fractures par le Massage et la Mobilisation, 1895." According to Landerer's method, described by Lumniczer,[3] the ends of the bone are to be replaced immediately after the fracture, and to be fixed in a firm dressing (such as plaster of Paris) until the disappearance of the swelling due to the injury; and when the soft provisional callus is fully formed (which takes place in from eight to fourteen days), the callus and the surrounding muscles are to be massaged twice a day—at first gently, but later more energetically; and the mobility of the neighbouring joints is to be kept up by active and passive movements. When this is done it is noticed that the callus, which during the first few days was soft and yielding, soon hardens and becomes strong, and the joints, at first somewhat stiff, rapidly recover their mobility. Eighty-nine cases of simple and four cases of compound fracture were thus treated at the Second Surgical Clinic at Budapest. This treatment is not to be recommended in comminuted fractures or in cases where there is a very great tendency to displacement of the ends of the bones.

**Upper Extremity.**—According to Routier[4] fractures of the clavicle should be treated by open operation and the fragments replaced in position when there is any risk of much subsequent swelling and serious deformity, and whenever it is impossible by ordinary means to overcome such displacement as would not only be unsightly but also interfere with the innervation of the upper extremity. This author reports a case of fractured clavicle with extreme deformity in a female aged 22 successfully treated by suturing. In three weeks the union was perfect and the linear cicatrix was small and hardly perceptible. There was not the least trace of deformity, the shoulders being symmetrical. Keener[5] reports a successful case of treatment by open incision for irreducible dislocation of the shoulder joint in a muscular man aged 22. The head of the bone was found deeply placed between the pectoralis major and minor. The capsule was not ruptured, but seemed to be pushed before the head of the bone, while two cord-like fibrous bands on each side of the anatomical neck seemed to effectually prevent reduction. After division of both the bands the bone was easily replaced. The wound healed by first intention, and the joint was as good as ever. The same joint had been dislocated in childhood.

A dislocation of the same bone without fracture in a patient 94 years of age was readily reduced by simple extension under chloroform by W. Lenton Heath.[6] Lejars[7] has operated with advantage upon two cases of badly-united fractures of the upper end of the humerus with deformity and loss of function. The uniting callus was removed and the fragments replaced and held in position by periosteal and fibrous sutures only, and in such cases an osseous suture may be required. Two inches and a half of comminuted fragments of the shaft of the humerus were removed by Wherry[8] in the case of an accidental gun-shot wound. The shot entered at the level of the axillary fold, but the axillary artery and nerves were uninjured. Stromeyer's cushion and shawl were the only retentive apparatus used, and good bony union resulted. Wherry reported a similar case many years ago in which Stromeyer's cushion was used, and recovery ensued with a useful limb. The results of treatment of injuries of the lower end of the humerus by flexion is recorded by J. Hutchinson, jun.,[9] in a paper read before the last meeting of the British Medical Association at Bristol. His own experience of the extension method in these cases has been the reverse of favourable. In subjects under 10 years of age the commonest form of injury is a partial detachment of the lower epiphysis, of which the author gives a figure. At the same meeting Professor Landerer[10] advocated his treatment (mentioned above) of these injuries, viz., by combining fixation or extension with early massage, use, and gymnastics. Professor Albert[11] showed at the Imperio-Royal Medical Society of Vienna a child suffering from external subluxation of the elbow, an affection frequently confounded with fractures of the epicondyle. Such subluxations are very difficult to reduce. Weinlechner had never met with a pure lateral subluxation in a child; as a rule, fracture of the epico ıdyle exists at the time, though unrecognised. Tescuner[12] showed before the New York Academy of Medicine an unusual

Humerus showing separation of the lower epiphysis united with lateral displacement.

fracture of the ulna and radius in a boy. The end of the lower fragment of the radius was in contact with the end of the upper fragment of the ulna. Four short splints, one on each of the four aspects of the forearm, were applied, and over these a longer splint with a webbing, extending two inches beyond the fingers, to give sufficient extension to prevent the fractured ends from slipping past one another. The result was perfect.

[1] Med. Week, Nov. 16, 1894. [2] Lucas Championnière, Traitement des fractures par le massage et la mobilisation, Paris, 1895, pp. 551. [3] Lancet, Oct. 27, 1894. [4] Epit Brit. M. J., Dec. 8, 1894, and Rev. d'Orthopédie, No. 6, 1894. [5] N. Y Med. Journal, Dec. 8, 1894. [6] Lancet, Oct. 27, 1894. [7] Revue de Chir., Aug. 10, 1894, pp. 636 and 642. [8] Lancet, Jan. 19, 1895. [9] Brit. Med. Journal, Nov. 3, 1894, p. 965. [10] Ibid, p. 968. [11] Med. Week, Dec. 14, 1894. [12] N. Y. Med. Record, Oct. 27, 1894, p. 537.

## Figures and Tables

**Figure f1:**